# Supplementary material for: Reciprocal Regulation Between Indoleamine 2,3-Dioxigenase 1 and Notch1 Involved in Radiation Response of Cervical Cancer Stem Cells
Source: Cancers (Basel). 2020 Jun 12;12(6):1547. doi: 10.3390/cancers12061547 (PMC7352771; doi:10.3390/cancers12061547)
Supplement: Supplementary file 1 [file cancers-12-01547-s001.pdf]

cancers

MDPI

Supplementary Materials: Reciprocal Regulation Between Indoleamine 2,3-Dioxigenase 1 and Notch1 Involved in Radiation Response of Cervical Cancer Stem Cells

Table S1. The primer sets used in this study.

For ChIP	
**Target site**	Primer sequence (5′ to 3′)
IDO1 promoter	
Forward	caagaatggcacacgctatg
Reverse	ttggccaggtaaggaatgag
Notch1 promoter	
Forward	caggaggaggacggtgac
Reverse	attgcccgagcacttgac
For qRT-PCR	
Target gene	Primer Sequence (5′ to 3′)
BMI1	
Forward	aatccccacctgatgtgtgt
Reverse	gctggtctccaggtaacgaa
Oct/4	
Forward	ggtccgagtgtggttctgta
Reverse	cgaggagtacagtgcagtga
Sox2	
Forward	aaccccaagatgcacaactc
Reverse	cggggccggtatttataatc

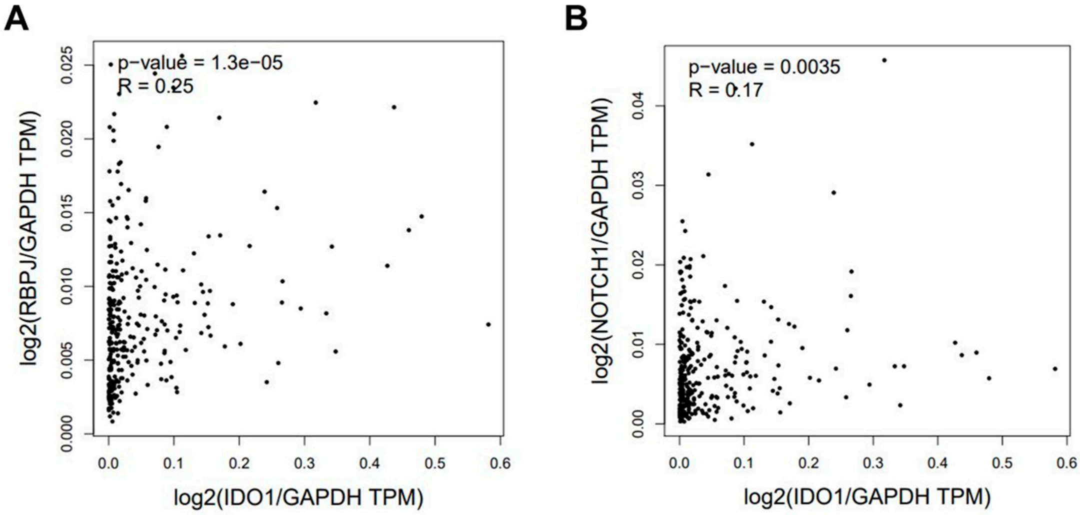

Figure S1. RBPJ or Notch1 expression was positively correlated with IDO1 among cervical cancer patients of TCGA **database.** RNA-Seq data of cervical cancer patients in TCGA database were analyzed and obtained from GEPIA website. (**A**) The correlation between RBPJ and IDO1. (**B**) The correlation between Notch1 and IDO1.

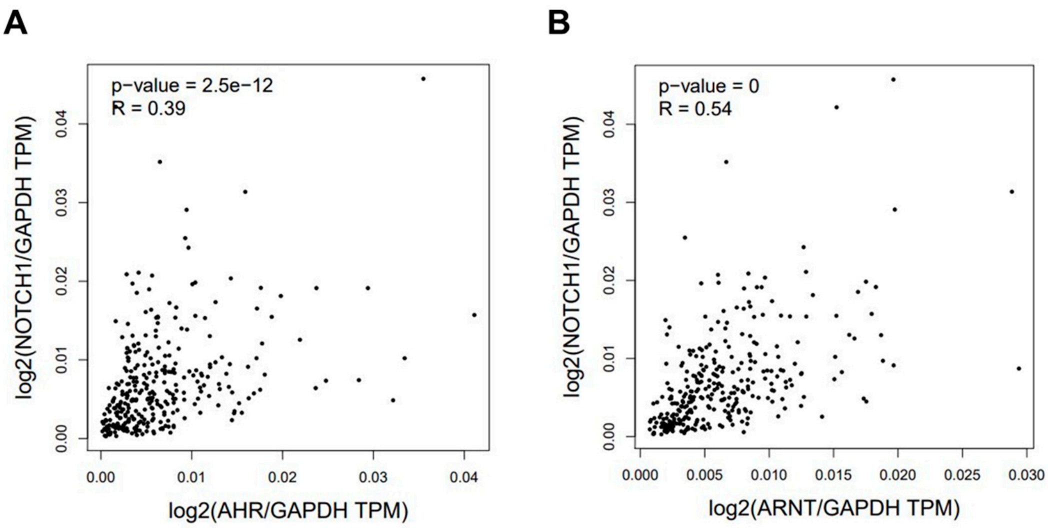

Figure S2. AhR or ARNT expression was positively correlated with Notch1 among cervical cancer patients of TCGA database. RNA-Seq data of cervical cancer patients in TCGA database were analyzed and obtained from GEPIA website. (**A**) The correlation between AhR and Notch1. (**B**) The correlation between ARNT and Notch1.

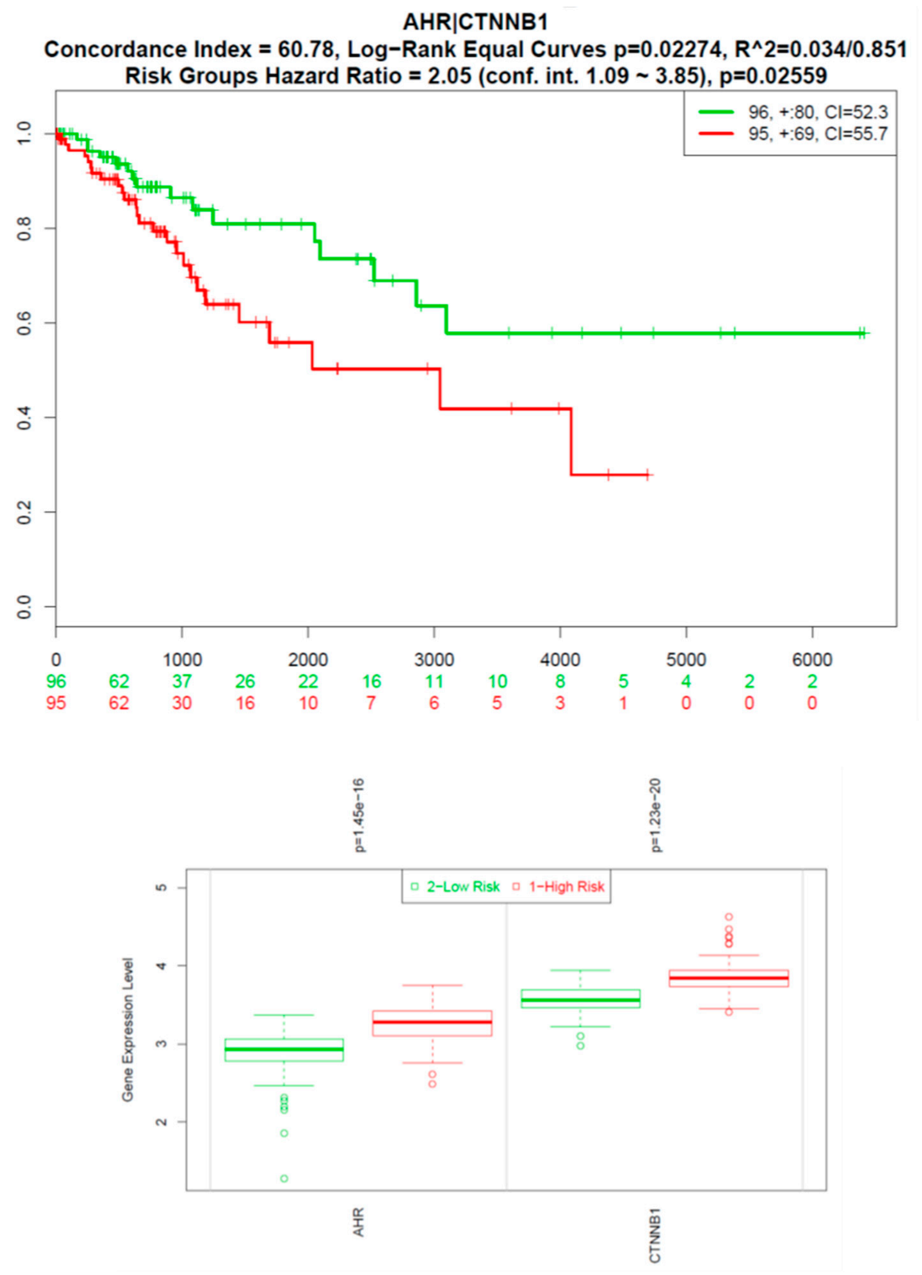

Figure S3. The combination of AhR and CTNNB1 expression level predicts survival outcome of cervical cancer patients**.** The RNA Seq data of cervical cancer patients in TCGA database was analyzed by SurExpress website. The combination of AhR and CTNNB1 expression served as a prognostic factor to patients' overall survival and both AhR or CTNNB1 displayed in significantly higher level in high risk group.

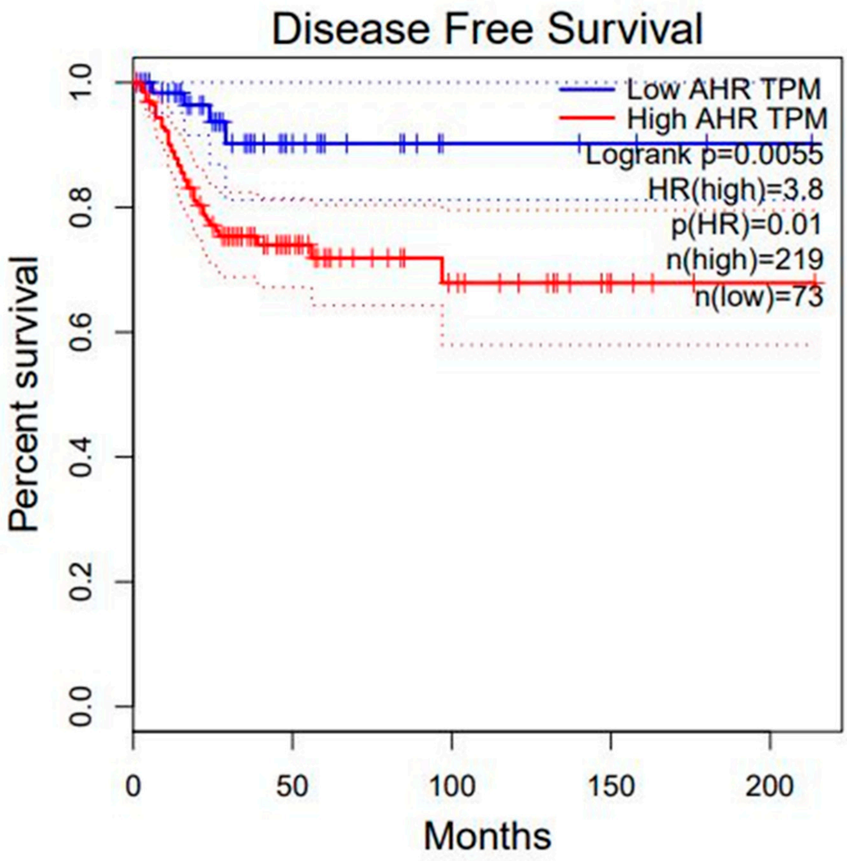

Figure S4. AhR expression level is positively correlated with shorter disease-free survival of cervical cancer patients in TCGA database. RNA-Seq and disease-free survival data of cervical cancer patients in TCGA database were analyzed and obtained from GEPIA website.

Fig. 1B

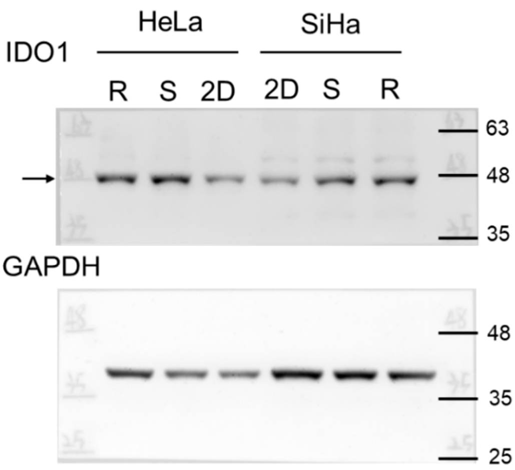

Fig. 1D

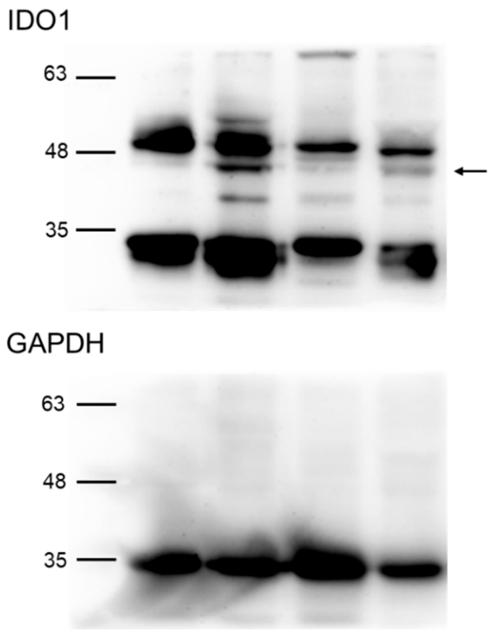

Fig. 2A

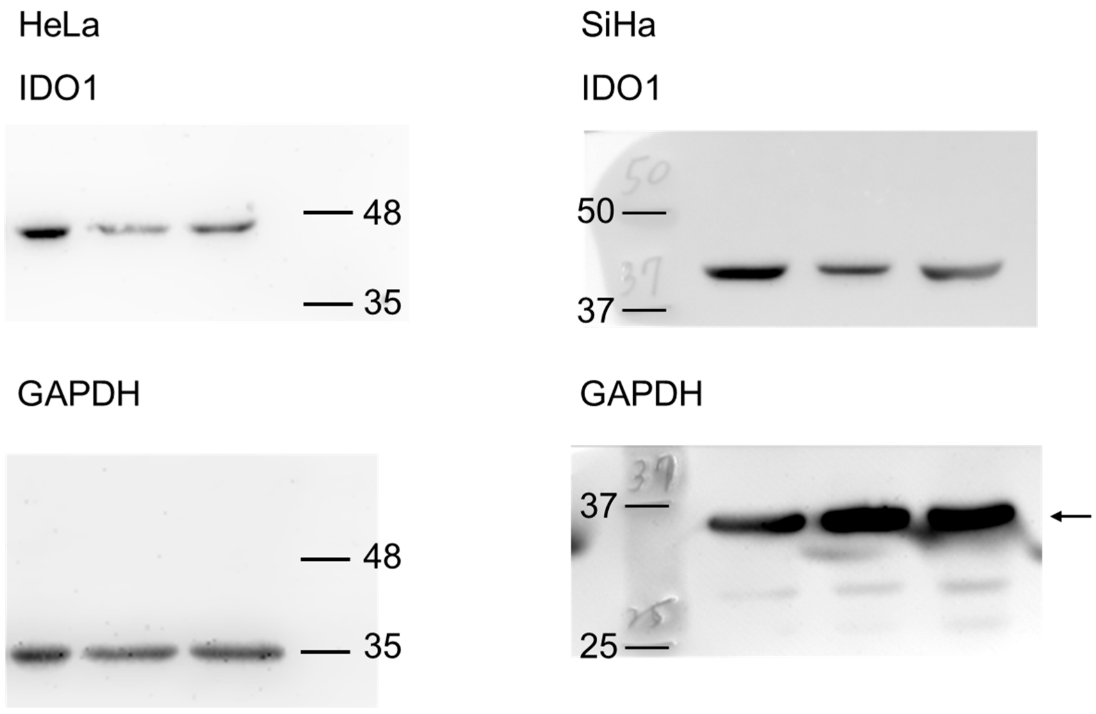

Fig. 3A

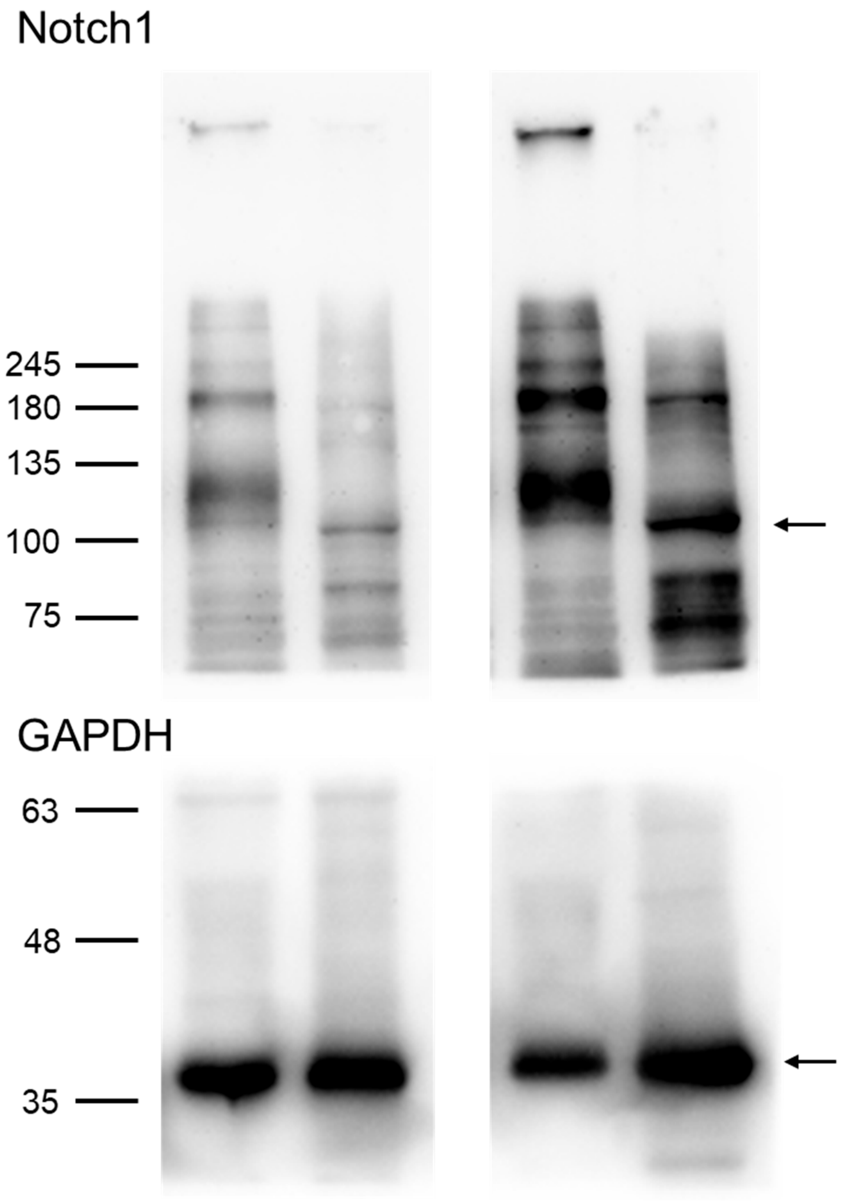

Fig. 3B

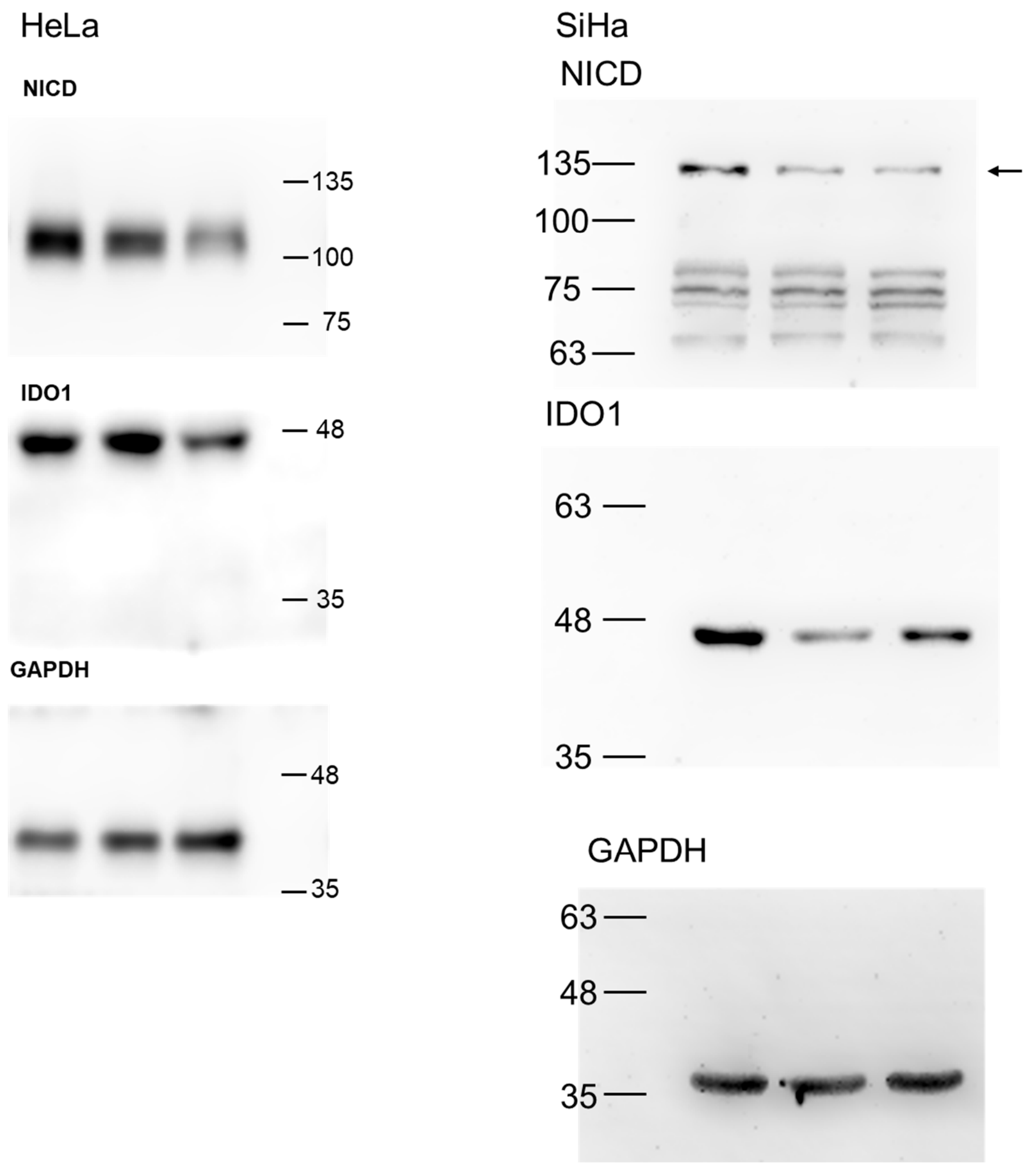
HeLa
NICD
135
100
75
IDO1
48
35
GAPDH
48
35
SiHa
NICD
135
100
75
63
IDO1
63
48
35
GAPDH
63
48
35

Fig. 3D

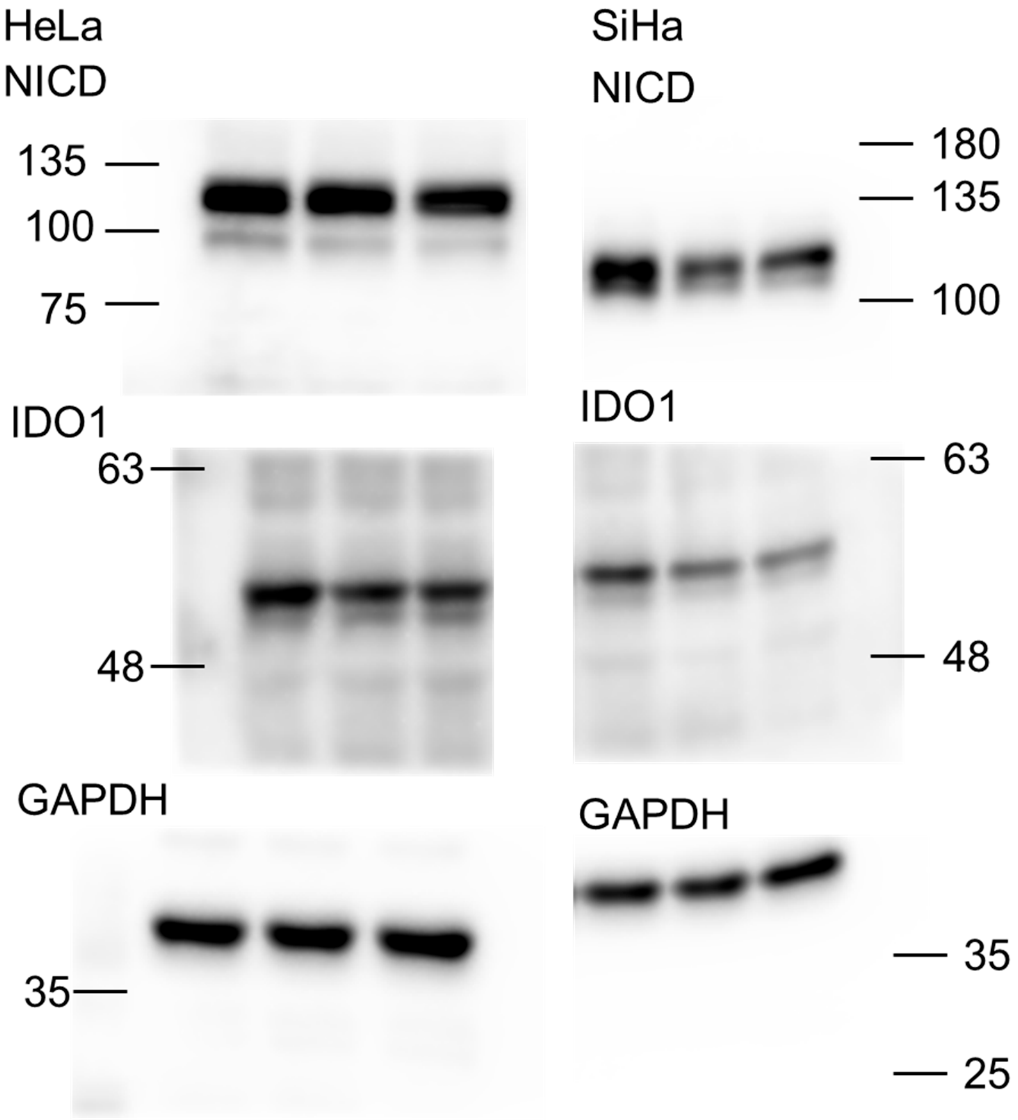
HeLa
NICD
135
100
75
IDO1
63
48
GAPDH
35
SiHa
NICD
180
135
100
IDO1
63
48
GAPDH
35
25

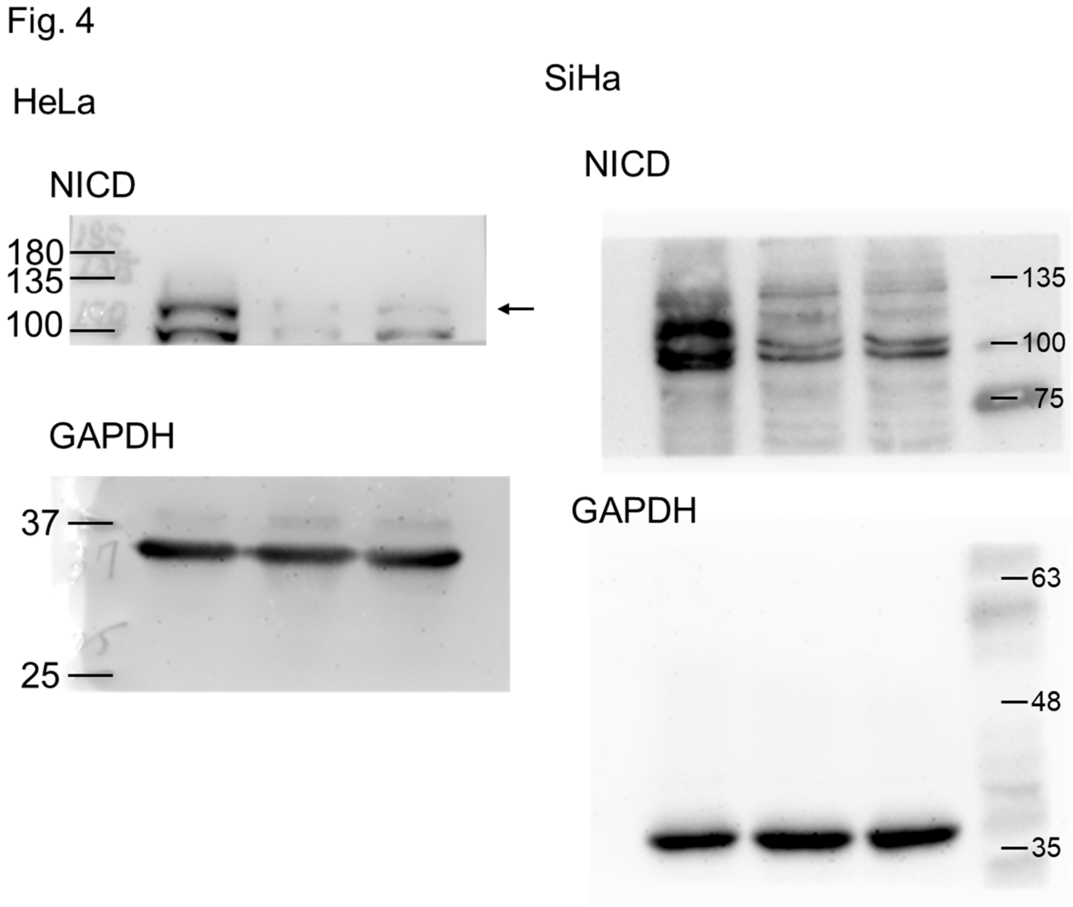
Fig. 4
HeLa
NICD
180
135
100
GAPDH
37
25
SiHa
NICD
135
100
75
GAPDH
63
48
35

Fig.5A

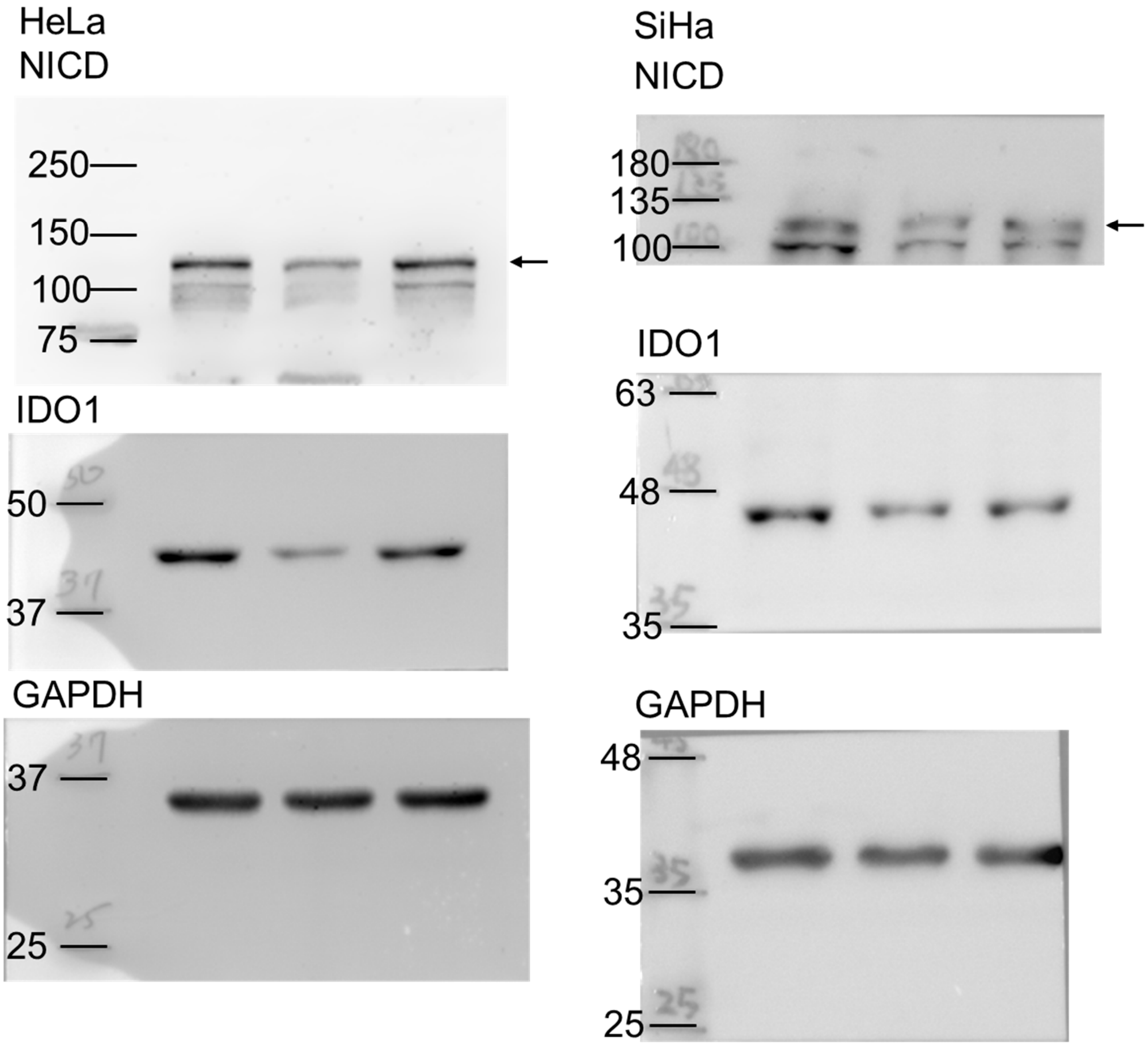
HeLa
NICD
250
150
100
75
IDO1
50
37
GAPDH
37
25
SiHa
NICD
180
135
100
IDO1
63
48
35
GAPDH
48
35
25

Fig. 5B

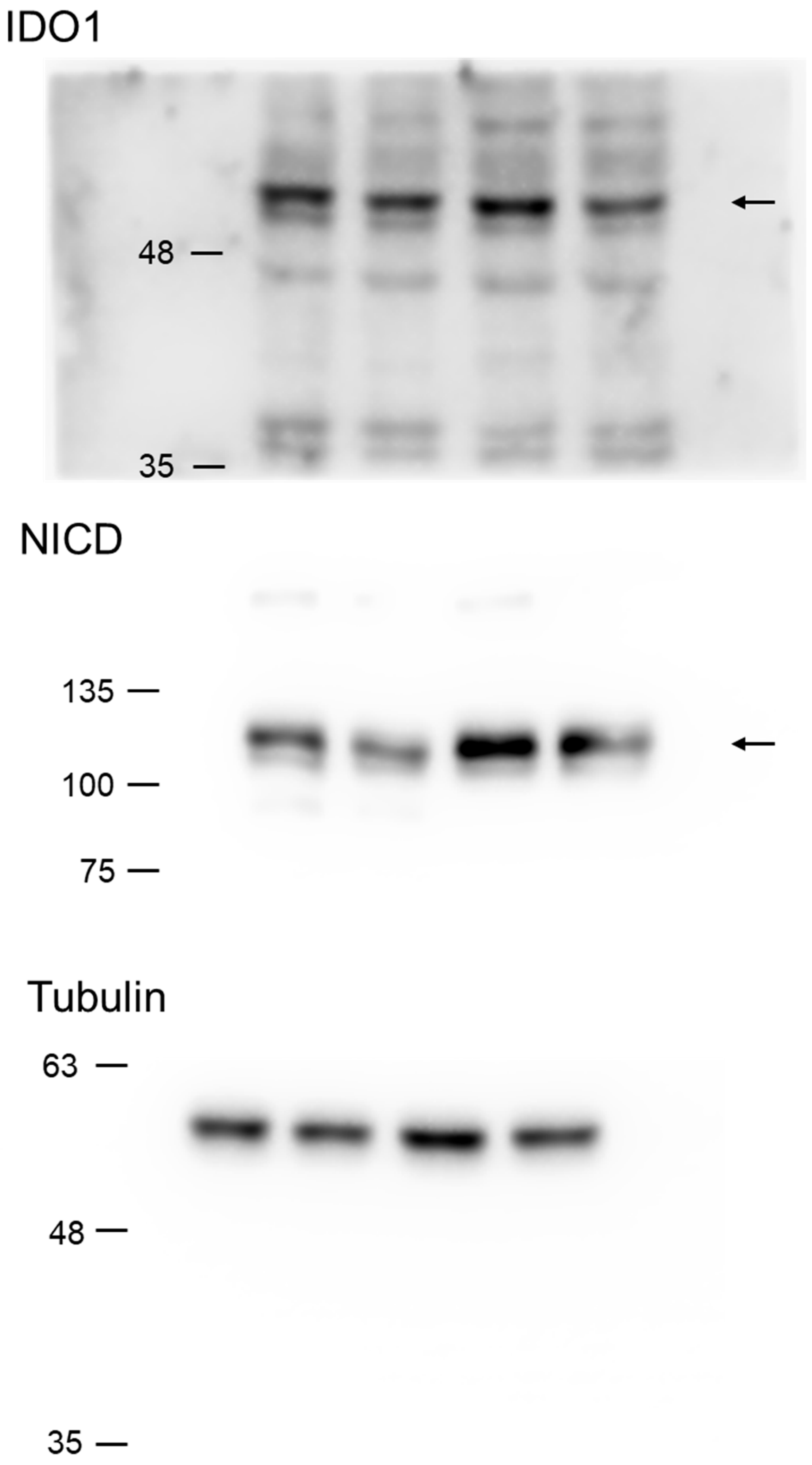

© 2020 by the authors. Submitted for possible open access publication under the terms and conditions of the Creative Commons Attribution (CC BY) license (http://creativecommons.org/licenses/by/4.0/).
